# Inhibition of Viability, Proliferation, Cytokines Secretion, Surface Antigen Expression, and Adipogenic and Osteogenic Differentiation of Adipose-Derived Stem Cells by Seven-Day Exposure to 0.5 T Static Magnetic Fields

**DOI:** 10.1155/2016/7168175

**Published:** 2016-01-06

**Authors:** Jian Wang, Bo Xiang, Jixian Deng, Darren H. Freed, Rakesh C. Arora, Ganghong Tian

**Affiliations:** ^1^Department of Vascular Surgery, Union Hospital, Tongji Medical College, Huazhong University of Science and Technology, 1277 Jiefang Street, Wuhan, Hubei 430022, China; ^2^National Research Council of Canada, 435 Ellice Avenue, Winnipeg, MB, Canada R3B 1Y6; ^3^Department of Physiology, Faculty of Medicine, University of Manitoba, 727 McDermot Avenue, Winnipeg, MB, Canada R3E 3P5; ^4^Department of Pharmacology and Therapeutics, Faculty of Medicine, University of Manitoba, 753 McDermot Avenue, Winnipeg, MB, Canada R3E 0T6; ^5^Division of Cardiac Surgery, University of Alberta Hospital, 8440-112 Street, Edmonton, AB, Canada T6G 2B7; ^6^Cardiac Science Program, Institute of Cardiovascular Science, St. Boniface General Hospital, 409 Tache Avenue, Winnipeg, MB, Canada R2H 2A6

## Abstract

After seven-day exposure to 0.5-Tesla Static Magnetic Field (SMF), Adipose-derived Stem Cells (ASCs) and those labeled by superparamagnetic iron oxide (SPIO) nanoparticles were examined for viability by methyl thiazol tetrazolium (MTT) assay, proliferation by cell counting and bromodeoxyuridine (BrdU) incorporation, DNA integrity by single cell gel electrophoresis, surface antigen by flow cytometry analysis, and the expression of cytokines and genetic markers by reverse transcription-PCR and underwent adipogenic and osteogenic differentiation assessed by quantifying related specific genes expression. The SMF slightly reduced cell viability and proliferation and inhibited the expression of CD49d, CD54, and CD73 but did not damage DNA integrity. The SMF slightly downregulated the expression of cytokines including Vascular Endothelial Growth Factor (VEGF), Insulin-like Growth Factor-1 (IGF-1), Transforming Growth Factor Beta 1 (TGF-*β*1), genetic markers comprising Stem Cell Antigen-1 (Sca1), Octamer-4 (Oct-4), ATP-binding Cassette Subfamily B Member 1 (ABCB1), adipogenic marker genes containing Lipoprotein Lipase (LPL), Peroxisome Proliferator-Activated Receptor Gamma (PPAR-*γ*), and osteogenic marker genes including Secreted Phosphor-protein 1 (SPP1) and Osterix (OSX). Exposure to 0.5 T SMF for seven days inhibited viability, proliferation, surface antigen expression, cytokine secretion, stem cell genetic marker expression, and adipogenic and osteogenic differentiation but did not affect the DNA integrity in ASCs with or without SPIO labeling.

## 1. Introduction

Stem cell transplantation is a promising therapeutic approach for ischemic Myocardial Infarction (MI), and its efficiency largely depends on cell homing, retention, and engraftment within the damaged myocardium. Poor cell retention is the major obstacle to achieving a satisfactory functional benefit irrespective of cell type and delivery routes [1–3]. Venous washout and the squeezing effect of cardiac contraction result in substantial cell loss during and immediately after delivery [4, 5]. Magnetic targeting techniques have been demonstrated to successfully enhance cardiac retention and functional benefits of “magnetic” iron-labeled cells via intramyocardial [6], intracoronary [7], intraventricular [8], and intravenous [9] routes to the ischemic hearts.

To track implanted cells* in vivo* in a noninvasive manner with repeated imaging is advantageous for animal models studies to clarify cell migration, tissue localization, and the longevity of cells following implantation. Superparamagnetic iron oxide (SPIO) particles can generate MRI contrast by disturbing the local Magnetic Field (MF) near excited spins, a property termed T_2_^*∗*^ relaxation. SPIO labeling does not affect the biological function and differentiation capacity of Stem Cells (SCs) [10, 11]. The cells labeled by SPIO can be visualized noninvasively* in vivo* using MRI serially over a period of weeks [12].

Exposition of SCs to MF is the fundamental principle of MRI tracking and magnetic targeting of implanted cells. Consequently, the influence of Static Magnetic Field (SMF) on biological function of SCs has been becoming a research topic of considerable interest. Most studies have focused primarily on the effects of MF on Bone Marrow Stem Cells (BMSCs). Recently, adipose tissue has emerged as a promising source of SCs for cell-based therapy due to the abundance in most of the population, easy and repetitive harvest with a minimum invasive procedure, and rapid proliferation [13]. Adipose-derived Stem Cells (ASCs) can differentiate into endothelial cells and smooth muscle cells, secrete multiple angiogenic cytokines, and eventually improve cardiac function after MI [14–16]. However, less information is available about the effect of SMF on the biological properties of ASCs.

SMF has been categorized according to its induction value as weak (<1 mT), moderate (1 mT to 1 T), strong (1–5 T), and ultrastrong (>5 T). Among these MF strengths, moderate intensity SMF can be easily applied in magnetic targeting or tracking technique and has been shown to affect physiological function in a variety of biological systems. Moreover, time-dependent changes in cell structural characteristics with days of exposure to moderate intensity SMF have been reported [17]. Brief exposure to moderate intensity SMF (two days) resulted in slight cellular alteration with return to baseline level within minutes following termination of exposure [17]. The present study was carried out to ascertain whether a prolonged exposure (seven days) to moderate intensity SMF (0.5 T) affect viability, proliferation, apoptosis, DNA structural integrity, surface antigen expression, angiogenic cytokine secretion, stem cell genetic marker expression, and adipogenic and osteogenic differentiation of ASCs and SPIO-labeled ASCs (_SPIO_ASCs).

## 2. Materials and Methods

This study was approved by the Institutional Review Board and Animal Care Committee of Huazhong University of Science and Technology and National Research Council of Canada.

### 2.1. Preparation of ASCs

Subcutaneous adipose tissue was obtained from the abdominal regions of inbred male Lewis rats. The excised adipose tissue was minced and digested with collagenase I (2 mg/mL, Worthington Biochemical Corp., Lakewood, NJ, USA) at 37°C for 20–30 min. The digested adipose tissue was filtered twice with a 100 *μ*m and then with a 25 *μ*m nylon membrane to eliminate the undigested fragments. The cellular suspension was centrifuged at 1000 ×g for 10 min. The cell pellets were resuspended at a density of 5 × 10^6^ cells/mL in Cell-Culture Medium (CCM) with Dulbecco's Modified Eagle's Medium/Ham's F-12 (DMEM/F12; HyClone Laboratories, Logan, UT) containing 15% fetal bovine serum (FBS; HyClone, Logan, UT) and cultivated for 24 h at 37°C in 5% CO_2_.

The ASCs were incubated for 2 days in a CCM containing 50 *μ*g/mL SPIO nanoparticles (Feridex; Bayer Healthcare Pharmaceuticals, Berlin, Germany; 100 mg/mL, 62 nm in diameter) and 6 *μ*g/mL protamine sulfate (a transfecting agent). Loading of SPIOs into ASCs was confirmed by Prussian blue staining.

### 2.2. Exposure of ASCs to the SMF

**Figure 1:**
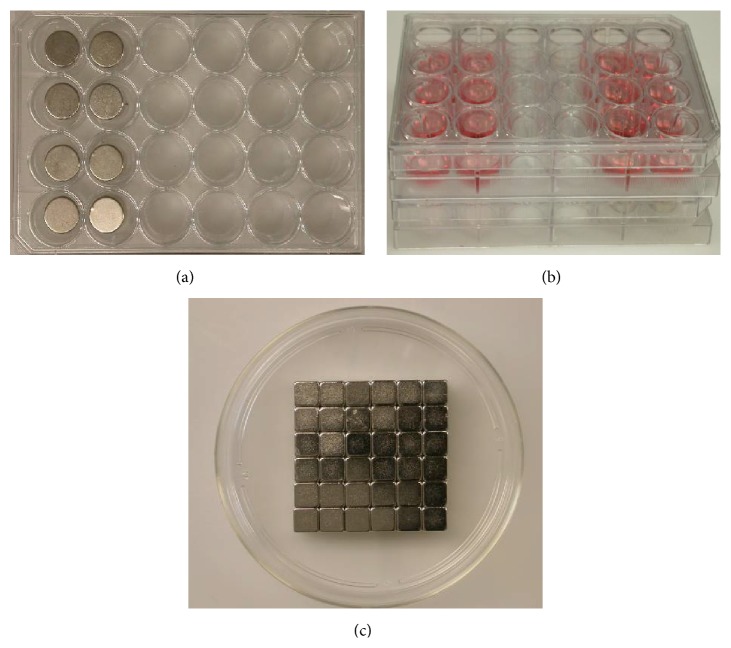
0.5 T SMF exposure system. (a) Eight circular magnets were embedded in the wells of the first and second columns on the 24- or 96-well plates. (b) The magnet-embedded plates were placed firmly at the bottom of the cell-cultured ones in a well-to-well fashion. (c) Cubic magnets were positioned below a Petri dish.

A 15 mm diameter (wide) and 1 mm thick neodymium-iron-boron (NdFeB) N50 grade disc or cubic magnet with the residual induction (Br) of 600 mT (Indigo Instruments, Waterloo, ON, Canada) was used to create the SMF. Circular magnets were inserted into each well of the first two adjacent columns on the 24- or 96-well plates (Figure 1(a)). ASCs and _SPIO_ASCs were seeded into the wells of the first and last two columns, respectively. The magnet-embedded plates were firmly placed below the cell-cultured ones in a well-to-well manner in order to expose the first two columns to SMF (Figure 1(b)). Moreover, cubic magnets (1 mm thick, 15 mm wide, Br = 600 mT, Indigo Instruments, Waterloo, ON, Canada) were positioned under a Petri dish (Figure 1(c)). The exposed and the control ASCs were kept in the same incubator but with at least 30 cm distance gap. There was no detectable field from the magnet around the control ASCs. The distance between the single layer of ASCs and the surface of the magnet was constant and identical. The SMF induction values at inner wall of well and flask were 0.506 ± 0.01 Tesla by a LakeShore Model 420 Gaussmeter (LakeShore Cryotronics, Westerville, OH, USA) in incubators at 37°C in 5% CO_2_ humidified atmosphere during the experiments. Magnetic induction values of the environmental geomagnetic field were about 5 orders of magnitude lower than those inside cell containers. All experiments were conducted in the conditions without heat generation.

The cells were cultured for a seven-day period with or without the continuous exposure of SMF. Following seven-day cultivation an aliquot of exposed and control cells were prepared for postexposure evaluation and induction differentiation. The 24-well plates were used for the proliferation rate measurement with an automated cell counter. The 96-well plates were used for cell viability and bromodeoxyuridine (BrdU) cell proliferation assays. The Petri culture dish was used for comet assay, apoptotic staining, flow cytometric assay, reverse transcription- (RT-) PCR, and induction differentiation analysis.

### 2.3. Cell Viability Assessment

ASC's viability was measured by the MTT [3-(4,5-methylthiazol-2-yl)-2,5-diphenyl-tetrazolium bromide] assay (Sigma-Aldrich, St. Louis, MO, USA). Briefly, a 5 mg/mL MTT salt solution was added to cells to give a final concentration of 2.5 mg/mL MTT. Cells were then incubated at 37°C for 1 h. The final formazan product was dissolved in dimethyl sulfoxide and light absorbance was measured at 570 nm. The amount of formazan was directly proportional to the number of live cells.

### 2.4. Cell Proliferation Assay

For Cell Counting Kit (CCK) assay, a Cell Counting Kit reagent (Sigma-Aldrich, St. Louis, MO, USA) was added to the well and incubated for 2 h. Absorbance value was measured at 450 nm using a microplate reader (Bio-Rad Laboratories, Hercules, CA, USA). For BrdU incorporation, BrdU labeling reagent (Roche Diagnostics, Indianapolis, IN, USA) was added to each well and cells were reincubated for 24 h at 37°C. After removing CCM, cells were fixed and DNA denatured by adding 200 mL of FixDenat (Roche Diagnostics, Indianapolis, IN, USA) for 30 min at room temperature. After removing FixDenat, anti-BrdU-POD working solution was added to each well and left at room temperature on a shaker for 90 min. Each well's absorbance was measured at 370 nm with a microplate reader (Bio-Rad Laboratories, Hercules, CA, USA).

### 2.5. Tunnel Staining

ASCs and _SPIO_ASCs exposed or unexposed to SMF were stained by Terminal Deoxynucleotidyl Transferase Mediated dUTP Nick End Labeling (TUNEL) with* In Situ* Cell Death Detection Kit (Roche Diagnostics, Indianapolis, IN, USA) according to the manufacturer's instructions. After TUNEL, the sections were counterstained for nuclei with 4′,6-diamidino-2-phenylindole (DAPI; Sigma-Aldrich, St. Louis, MO, USA). The TUNEL or DAPI positive nuclei were counted in three random fields under a Zeiss Axiophot fluorescence microscope (Carl Zeiss, Oberkochen, Germany).

### 2.6. Single Cell Gel Electrophoresis (Comet Assay)

Five microliters of cells was mixed with 75 *μ*L low melting point agarose (0.5% at 37°C) and pipetted onto slides precoated with 1% normal melting point agarose in 2 parallels. This suspension was maintained at 4°C until solidification of the agarose. The slides were immersed in freshly prepared cold cell lyses buffer for 24 h at 4°C and then placed in a horizontal electrophoresis unit (Bio-Rad Laboratories, Hercules, CA, USA) containing freshly prepared electrophoresis buffer. The electrophoresis was performed at 25 V, 300 mA for 30 min. The DNA was stained with 50 *μ*L of 0.5 mg/mL ethidium bromide. Using a fluorescence microscope with wavelengths of 535 and 590 nm, photography of a representative image of each slide was taken.

### 2.7. Flow Cytometry Analysis

The cells were fixed for 10 min in 1% paraformaldehyde. After washing twice with PBS, cells were incubated with primary antibodies at room temperature for 30 min. The antibodies used were as follows: fluorescein isothiocyanate- (FITC-) conjugated anti-rat CD49d, CD54, and CD73 (Invitrogen, Carlsbad, CA, USA). Flow cytometric analysis was performed on a fluorescence-activated cell sorter (BD Biosciences, CA, USA).

### 2.8. Adipogenic and Osteogenic Differentiation

Before performing the experiments, ASCs and _SPIO_ASCs were cultured for seven days with or without SMF exposure. Adipogenic differentiation was induced by incubation of the cells for 12 days in DMEM containing 5% FBS, 10 *μ*M bovine insulin, 250 isobutyl-methylxanthine, 200 *μ*M indomethacin, 1 *μ*M dexamethasone, 5 *μ*g/mL streptomycin, and 5 U/mL penicillin. Osteogenic induction was carried out by cultivating the ASCs for 28 days in DMEM containing 10% FBS, 10 mM *β*-glycerophosphate, 50 *μ*M ascorbate-2-phosphate, 10 nM 1,25(OH)_2_ vitamin D3, 5 *μ*g/mL streptomycin, and 5 U/mL penicillin.

### 2.9. Oil-Red-O Staining

After 12-day adipogenic induction, the cells were fixed in 4% paraformaldehyde for 30 min at room temperature and washed twice with distilled water. Then they were stained in Oil-red-O at a ratio of 60% stock solution (0.5% weight/volume in isopropanol) to 40% distilled water for 15 min. The cells were examined under light microscopy (TE2000-U; Nikon, Tokyo, Japan). Oil-red-O levels in the stained cells were determined by measuring the optical density (OD) with a spectrophotometer (NanoDrop Technologies, Wilmington, DE, USA) at 495 nm (OD_450_).

### 2.10. Alkaline Phosphatase Staining

After 28-day osteogenic induction, the cells were fixed in 4% paraformaldehyde for 30 min at room temperature and washed twice with distilled water. Then they were stained in 1 mL Michaelis buffer containing 10 mg nitroblue tetrazolium chloride/bromo-4-chloro-3-indolyl phosphate toluidine salt (NBT/BCIP) for 20 min at 37°C. The NBT/BCIP undergoes an oxidation/reduction reaction after dephosphorylation by alkaline phosphatase (ALP), giving rise to an insoluble brown product. The stained cells were then examined under a light microscope. The brown product was also quantified with a spectrophotometer at 465 nm.

### 2.11. RT-PCR

Total RNA from the ASCs was extracted using the TRIzol Reagent (Invitrogen, Carlsbad, CA, USA) protocol. One microgram of RNA was reversely transcribed using SuperScript III reverse transcriptase (Invitrogen, Carlsbad, CA, USA). cDNA was used as a template for PCR amplification. Glyceraldehyde-3-phosphate dehydrogenase (GAPDH) was used as an internal control. The genes for adipogenic differentiation were Lipoprotein Lipase (LPL) and Peroxisome Proliferator-Activated Receptor Gamma (PPAR-*γ*). The genes for osteogenic differentiation were the Secreted Phosphor-protein 1 (SPP1) and Osterix (OSX).

Expressions of cytokines Vascular Endothelial Growth Factor (VEGF), Transforming Growth Factor Beta 1 (TGF-*β*1), Insulin-like Growth Factor-1 (IGF-1), and stem cell-specific marker Octamer-4 (Oct-4), Stem Cell Antigen-1 (Sca1), and ATP-binding Cassette Subfamily B Member 1 (ABCB1) were also determined. Sequences for all the primers used in this study are detailed in Table 1.

### 2.12. Statistical Analysis

All data are expressed as means ± standard deviation. Statistical significance was evaluated with unpaired Student's *t*-test for comparisons between two means, with ANOVA for more than two means. Data were considered significant when the *P* value was <0.05. All statistical analyses were performed with SPSS version 12.0 software (SPSS, Inc., Chicago, IL, USA).

## 3. Results

### 3.1. The SMF Captures “Magnetic” _SPIO_ASCs* In Vitro*

**Figure 2:**
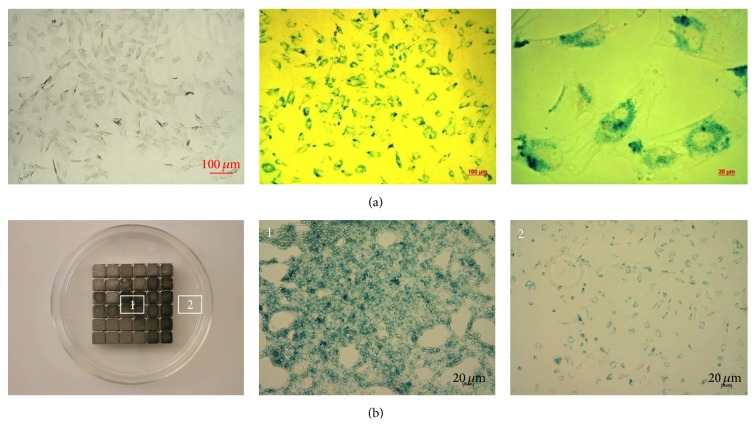
Magnetic accumulation of the _SPIO_ASCs* in vitro*. (a) There was heavy perinuclear distribution of blue-stained SPIOs in _SPIO_ASCs with the fibroblast-like morphology. (b) The “magnetic” _SPIO_ASCs were substantially attracted to the site where the magnet was positioned. Scale bar represents 20 *μ*m, 50 *μ*m, or 100 *μ*m.

The ASCs appeared as fibroblast-like morphology under a phase-control microscopy. The Prussian blue staining of the ASCs showed intracytoplasmic iron inclusions as dense blue-stained vesicles, and the magnetic SPIOs were distributed evenly around the nucleus of the ASCs as spherical shell (Figure 2(a)).

The _SPIO_ASCs were resuspended at the cell-culture dish, and then the magnet was placed behind the outer wall. After seven days of culture, a distinct cell population was focally formed on the inner dish wall. Conversely, the smaller cell population was present at place one centimeter away from the magnet (Figure 2(b)).

### 3.2. The SMF Decreases the Viability and Proliferation of ASCs and _SPIO_ASCs

**Figure 3:**
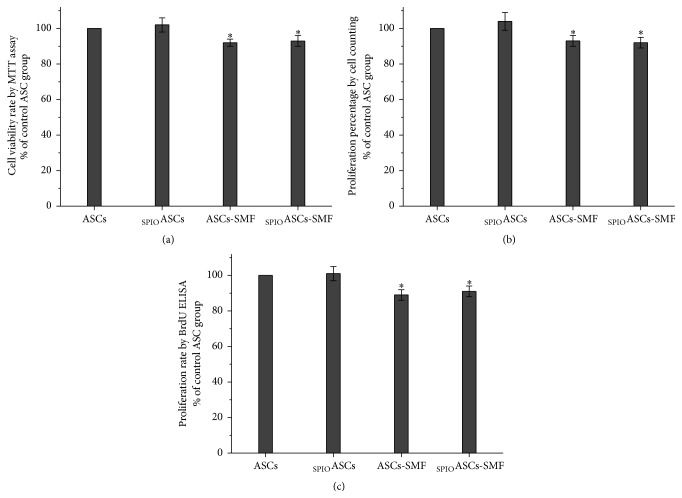
Effect of seven-day exposure to 0.5 T SMF on cell viability and proliferation in ASCs and _SPIO_ASCs. (a)–(c) Cell proliferation was assessed by cell counting (b) and BrdU assay (c). The cell viability (a) and proliferation rate ((b)-(c)) were significantly lower in the ASCs-SMF and _SPIO_ASCs-SMF than in ASCs and _SPIO_ASCs. ^*∗*^ *P* < 0.05 versus ASCs or _SPIO_ASCs.

Cell viability and proliferation rate have been normalized to values measured from the control ASCs, defined as 100%. Cell viability rates were 100%, 102% ± 4%, 92% ± 2%, and 93% ± 2% in ASCs, _SPIO_ASCs, ASCs-SMF, and _SPIO_ASCs-SMF, respectively (Figure 3(a)). Cell proliferation percentages measured by cell counting were 100%, 104% ± 5%, 93% ± 3%, and 92% ± 3% in ASCs, _SPIO_ASCs, ASCs-SMF, and _SPIO_ASCs-SMF, respectively (Figure 3(b)). Similarly, cell proliferation rates assessed by BrdU ELISA were 100%, 101% ± 4%, 89% ± 3%, and 91% ± 3% in ASCs, _SPIO_ASCs, ASCs-SMF, and _SPIO_ASCs-SMF, respectively (Figure 3(c)). The SMF-treated cells exhibited statistically significant decline in viability and proliferation in comparison with the control cells unexposed to SMF after seven-day exposure.

### 3.3. The SMF Inhibits the Expression of Surface Antigens in ASCs and _SPIO_ASCs

**Figure 4:**
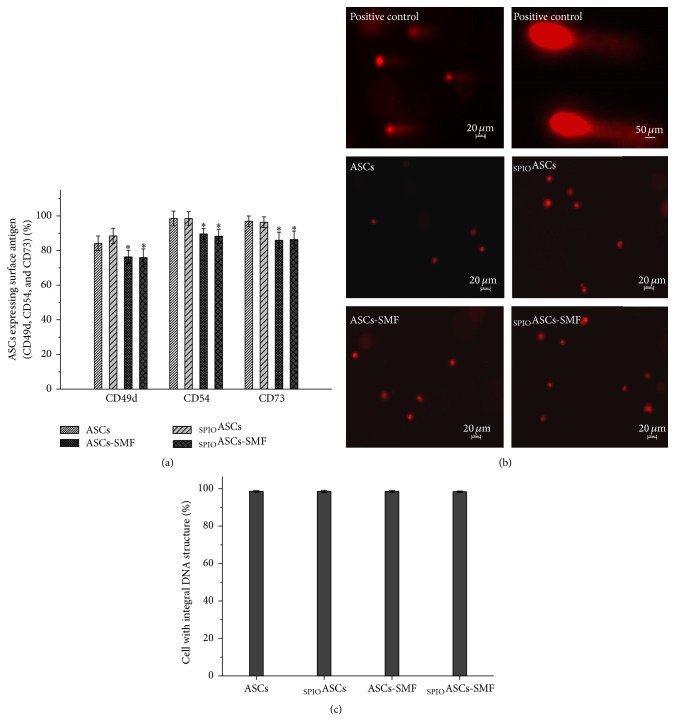
Effect of seven-day exposure to 0.5 T SMF on surface antigen profile and DNA integrity in ASCs and _SPIO_ASCs. (a) The percentage of cells expressing CD49d, CD54, and CD73 was significantly lower in the ASCs-SMF and _SPIO_ASCs-SMF than in the ASCs and _SPIO_ASCs. (b) Representative comet assay result. DNA damage with comet tail was not observed at four groups of ASCs. (c) The percentages of cell with integral DNA structure did not differ among ASCs, _SPIO_ASCs, ASCs-SMF, and _SPIO_ASCs-SMF. Scale bar represents 20 *μ*m or 50 *μ*m. ^*∗*^ *P* < 0.05 versus ASCs or _SPIO_ASCs.

The percentage of cells expressing CD49d, CD54, and CD73 after seven days of culture was reduced in ASCs-SMF and _SPIO_ASCs-SMF compared to ASCs and _SPIO_ASCs (76.35% ± 3.81% and 75.91% ± 5.05% versus 84.21% ± 4.21% and 88.47% ± 4.42% for CD49d; 89.57% ± 3.27% and 88.19% ± 4.15% versus 98.55% ± 4.27% and 98.50% ± 3.92% for CD54; 85.86% ± 4.81% and 86.27% ± 4.96% versus 96.92% ± 3.04% and 96.36% ± 3.12% for CD73, resp.) (Figure 4(a)).

### 3.4. The SMF Does Not Cause the DNA Damage and Apoptosis of ASCs and _SPIO_ASCs

The presence of comet tail was indicative of DNA double-strand breaks. Seven-day exposure to 0.5 T SMF was not associated with the DNA damage with comet tail in four cell groups (Figure 4(b)). The percentages of cell with integral DNA structure were not significantly different among the ASCs (98.49% ± 0.44%), _SPIO_ASCs (98.49% ± 0.62%), ASCs-SMF (98.51% ± 0.58%), and _SPIO_ASCs-SMF (98.36% ± 0.35%) (Figure 4(c)). Seven-day exposure to a 0.5 T SMF did not result in the apoptosis of ASCs and _SPIO_ASCs (data not shown).

### 3.5. The SMF Depresses Cytokines Secretion and Stem Cell Marker Expression in ASCs and _SPIO_ASCs

**Figure 5:**
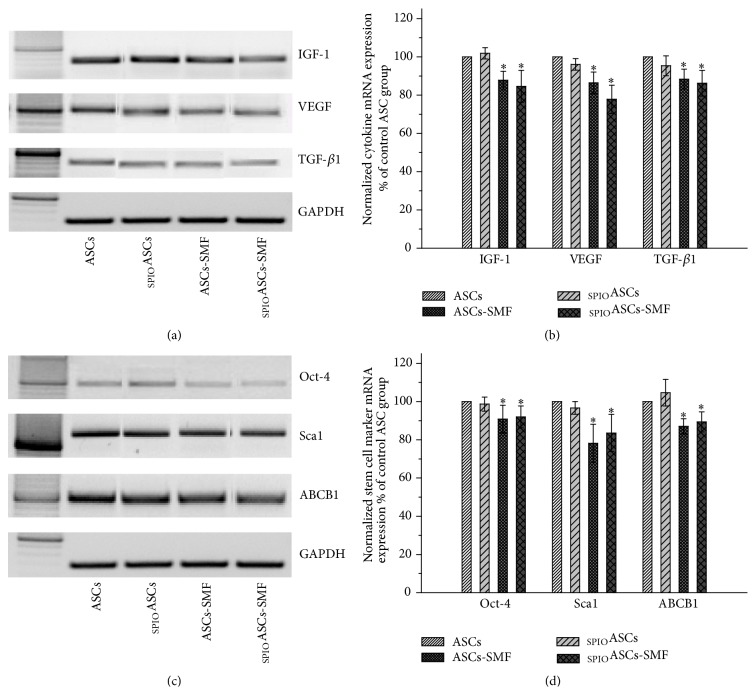
Effect of seven-day exposure to 0.5 T SMF on cytokine secretion and stem cell genetic marker expression in ASCs and _SPIO_ASCs. (a) Representative results of RT-PCR for IGF-1, VEGF, and TGF-*β*1. (b) Quantification after normalization to levels from control ASCs. The expression of mRNA for IGF-1, VEGF, and TGF-*β*1 was significantly lower in the ASCs-SMF and _SPIO_ASCs-SMF than in ASCs and _SPIO_ASCs. (c) Representative results of RT-PCR for Sca1, ABCB1, and Oct-4. (d) Quantification after normalization to levels from control ASCs. The expression of mRNA for Sca1, ABCB1, and Oct-4 was significantly lower in the ASCs-SMF and _SPIO_ASCs-SMF than in ASCs and _SPIO_ASCs. ^*∗*^ *P* < 0.05 versus ASCs or _SPIO_ASCs.

Figure 5(a) showed the representative results of RT-PCR for angiogenic cytokines including IGF-1, VEGF, and TGF-*β*1. PCR product band intensities, relative to GAPDH, have been normalized to values measured from the control ASCs, defined as 100%. ASCs-SMF and _SPIO_ASCs-SMF expressed the lower levels of IGF-1 (0.87- and 0.84-fold lower, resp.), VEGF (0.80- and 0.77-fold lower, resp.), and TGF-*β*1 (0.88- and 0.86-fold lower, resp.), compared with ASCs and _SPIO_ASCs (Figure 5(b)).

Figure 5(c) showed the representative results of RT-PCR for stem cell markers Sca1, ABCB1, and Oct-4. ASCs-SMF and _SPIO_ASCs-SMF expressed the lower levels of Sca1 (0.78- and 0.83-fold lower, resp.), ABCB1 (0.87- and 0.89-fold lower, resp.), and Oct-4 (0.90- and 0.91-fold lower, resp.), compared with ASCs and _SPIO_ASCs (Figure 5(d)).

### 3.6. The SMF Inhibits the Adipogenic Differentiation of ASCs and _SPIO_ASCs

**Figure 6:**
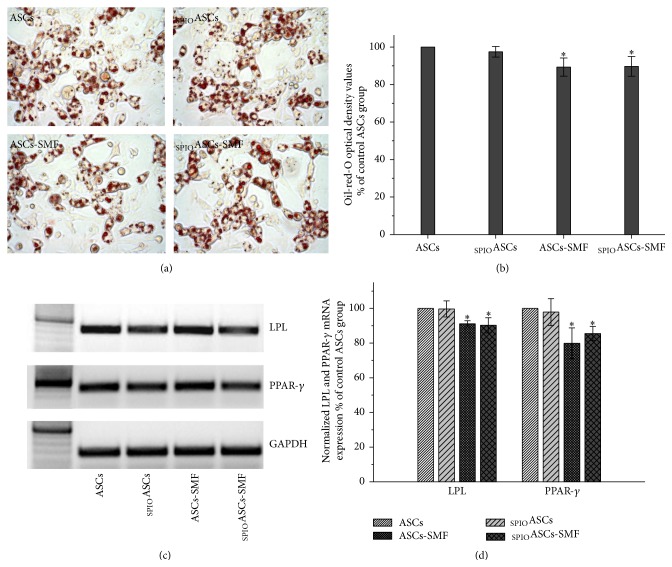
Effect of seven-day exposure to 0.5 T SMF on adipogenic differentiation in ASCs and _SPIO_ASCs. (a) Representative results of histochemical staining for Oil-o-red. (b) Quantification after normalization to levels from control ASCs. The Oil-o-red optical density value was significantly lower in the ASCs-SMF and _SPIO_ASCs-SMF than in ASCs and _SPIO_ASCs. (c) Representative results of RT-PCR for LPL and PPAR-*γ*. (d) Quantification after normalization to levels from control ASCs. The expression of mRNA for LPL and PPAR-*γ* was significantly lower in the ASCs-SMF and _SPIO_ASCs-SMF than in ASCs and _SPIO_ASCs. Scale bar represents 100 *μ*m. ^*∗*^ *P* < 0.05 versus ASCs or _SPIO_ASCs.

Histological staining showed strong Oil-red-O staining of the cytoplasmic lipid droplet (Figure 6(a)). Oil-red-O OD values were normalized to the level from the control ASCs defined as 100%. ASCs-SMF and _SPIO_ASCs-SMF had lower Oil-red-O OD value (0.89-fold and 0.89-fold lower, resp.) compared with ASCs and _SPIO_ASCs (Figure 6(b)).

Figure 6(c) showed the representative results of RT-PCR for adipogenic-related genes LPL and PPAR-*γ*. PCR product band intensities, relative to GAPDH, have been normalized to values measured from the control ASCs, defined as 100%. ASCs-SMF and _SPIO_ASCs-SMF expressed slightly lower levels of LPL (0.91- and 0.90-fold lower, resp.) and PPAR-*γ* (0.79- and 0.85-fold lower, resp.) compared with ASCs and _SPIO_ASCs (Figure 6(d)).

### 3.7. The SMF Inhibits the Osteogenic Differentiation of ASCs and _SPIO_ASCs

**Figure 7:**
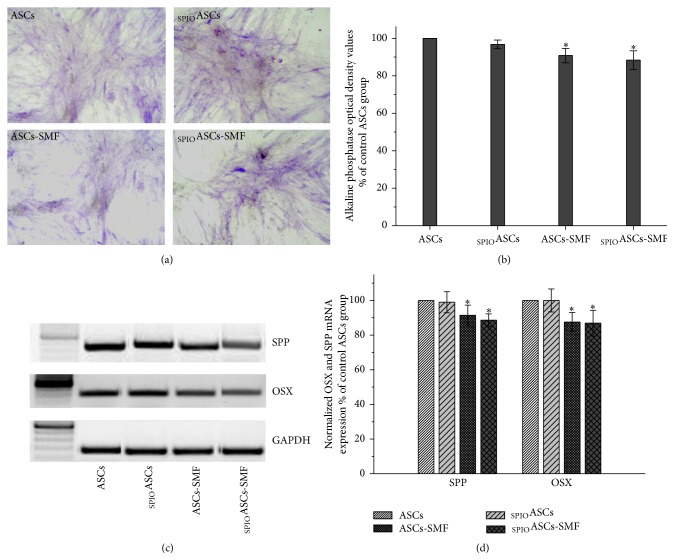
Effect of seven-day exposure to 0.5 T SMF on osteogenic differentiation in ASCs and _SPIO_ASCs. (a) Representative results of histochemical staining for alkaline phosphatase. (b) Quantification after normalization to levels from control ASCs. The alkaline phosphatase optical density value was significantly lower in the ASCs-SMF and _SPIO_ASCs-SMF than in ASCs and _SPIO_ASCs. (c) Representative results of RT-PCR for SPP and OSX. (d) Quantification after normalization to levels from control ASCs. The expression of mRNA for SPP and OSX was significantly lower in the ASCs-SMF and _SPIO_ASCs-SMF than in ASCs and _SPIO_ASCs. Scale bar represents 100 *μ*m. ^*∗*^ *P* < 0.05 versus ASCs or _SPIO_ASCs.

Histological staining showed strong ALP staining (Figure 7(a)). ALP OD values were normalized to the level from the control ASCs, defined as 100%. ASCs-SMF and _SPIO_ASCs-SMF had lower ALP OD value (0.90-fold and 0.88-fold lower, resp.) compared with ASCs and _SPIO_ASCs (Figure 7(b)).

Figure 7(c) showed the representative results of RT-PCR for osteogenic-related genes SPP and OSX. PCR product band intensities, relative to GAPDH, have been normalized to values measured from the control ASCs, defined as 100%. ASCs-SMF and _SPIO_ASCs-SMF expressed lower levels of OSX (0.87- and 0.86-fold lower, resp.) and SPP (0.91- and 0.88-fold lower, resp.) compared with ASCs and _SPIO_ASCs (Figure 7(d)).

## 4. Discussion

We have demonstrated that extended exposure to a 0.5 T SMF inhibits the cell viability and proliferation of ASCs with or without SPIO labeling. Our results are consistent with previous studies showing that SMF exposure decreases the cell proliferation. Marędziak et al. reported that seven-day exposure to a 0.5 T SMF significantly reduced the proliferation rate of ASCs of canine origin [18]. Javani Jouni et al. demonstrated that increasing of intensity (>15 mT) and time of SMF exposure (>96 hours) significantly decreased the viability percent and proliferation rate in BMSCs [19]. Rosen and Chastney found that a two-week exposure to a 0.5 T SMF was associated with a 37% decline in growth in GH3 cells with persistence for two weeks after the end of exposure [20]. When the exposure was extended to four weeks, the decline in growth was 51% with persistence for three weeks following removal from the magnet [20]. Raylman et al. reported that a 64 h exposure to a 7 T SMF reduced the viable cell number and inhibited the growth of three human tumor cell lines including HTB63 (melanoma), HTB 77 IP3 (ovarian carcinoma), and CCL 86 (lymphoma) [21]. There was no evidence that altered cell cycle and DNA strand break should be responsible for the magnetically induced slowed growth rate [21].

We found that SMF exposure does not have a harmful effect on DNA of ASCs and _SPIO_ASCs over seven days of exposure. The result is fairly consistent with those previously published papers. Amara et al. showed that SMF exposure (250 mT, during 3 h) did not cause DNA damage in THP1 cells [22]. Kubinyi et al. examined the effect of SMF on the DNA damage in leukocytes with exposures of homogeneous and inhomogeneous SMF for 1, 4, and 18 h, and they also could not identify any change in the DNA damage [23]. Reddig et al. demonstrated that exposure of human peripheral blood mononuclear cells to 7 T SMF did not induce DNA double-strand breaks [24].

In the study, the percentage of cells expressing surface antigen (CD49d, CD54, and CD73) was reduced in the ASCs and _SPIO_ASCs exposed to the SMF compared with the control ASCs and _SPIO_ASCs. In another experiment with human peripheral blood mononuclear cells, exposure for 2 h to a 0.5 T SMF resulted in a significantly reduced percentage of cells expressing CD69 after 24 h of* in vivo* culture [25]. Kim et al. reported that the expression levels of CD73, CD90, and CD105 were decreased in BMSCs exposed to a frequency of 45 Hz and an induction value of 1 mT EMF twice every 8 hours per day for 7 days [26].

We also showed that exposure to a 0.5 T SMF up to seven days inhibited the release of angiogenic cytokines (VEGF, IGF-1, and TGF-*β*1) and the expression of stem cell markers (Oct-4, Sal-1, and ABCB1) in ASCs and _SPIO_ASCs. The present study is the first one to investigate whether the SMF exposure impacts the cytokine and stem cell marker expression of ASCs and _SPIO_ASCs. Previously, Gruchlik et al. reported that SMF exposure (300 mT, 24 h) inhibited the Interleukin- (IL-) 6 secretion in normal human colon myofibroblasts [27]. Vergallo et al. demonstrated that exposure to a strong SMF (1.4 T) for up to 24 hours had a significant inhibitory effect on the release of proinflammatory cytokines IL-6, IL-8, and INF-*α* from macrophage [28]. Aldinucci et al. reported that exposure to a strong SMF (4.75 T) for 24–48 hours significantly decreased the production of IL-2 in human peripheral blood mononuclear cells [29].

In this study, moderate intensity SMF inhibited ASCs' adipogenic differentiation, which was shown by reducing adipocyte-specific expression of LPL and PPAR-*γ* and by decreasing the number of lipid droplets. This study also demonstrated that seven-day exposure to 0.5 T SMF reduced the ALP activity and osteogenic marker genes such as SPP and OSX and inhibited the osteogenic differentiation of ASCs. With respect to effects of moderate intensity SMF on ASCs' induction differentiation, published reports are conflicting. Schäfer et al. reported that exposition to 0.6 T SMF leads to a reduced expression ALP under osteogenic induction, indicating an impairment of osteogenic differentiation of BMSCs and SPIO-labeled BMSCs [30]. However, they also reported that exposition to 0.6 T SMF over 24 hours significantly promoted the expression of LPL and PPAR-*γ* in BMSCs and SPIO-labeled BMSCs, indicating an enhancement of adipogenesis of BMSCs under the exposure of SMF [30]. For the most part, our result is contrary to previous studies showing that MF exposure accelerates the osteogenic differentiation of SCs. Kim et al. showed that moderate intensity SMF, especially at 15 mT, promoted osteoblastic differentiation of MSCs, by upregulating genes associated with mineralization and calcium-banding proteins [31]. Amin et al. reported that a 0.4 T SMF applied for 14 days elicited a strong chondrogenic differentiation in cultured BMSCs [32].

The influence of MF on living cells depends on cell type, cell line, strength of the MF, and time of exposure. Increasing of SMF intensity or exposure time was associated with progressive decline in proliferation or viability rate in SCs and cancer cells [19, 20]. This discrepancy in cell type, intensity of SMF, and exposure time might explain the difference between our findings and previous ones. This study found that the 0.5 T SMF exposure for seven days could impair the biological properties and beneficial function of ASCs and _SPIO_ASCs. The SMF induction value and exposure time are two critical factors to affect the biological behavior of SCs. Magnetic targeting techniques have been reported to enhance cell retention, engraftment, and functional benefit of iron-labeled stem cells by 10-minute or 24-hour exposure to 1.3 T SMF (6, 7), 24-hour exposure to 0.1 T SMF (8), and 24-hour exposure to 0.6 T SMF (9) in infracted hearts. Further optimization is needed to identify the best magnetic strength and duration for effective targeting. However, this study indicated that simultaneously increasing SMF strength and exposure time might enhance the targeting effect but impair the biological activity and therapeutic efficacy of implanted SCs during magnetic targeting.

## 5. Conclusions

In summary, exposure to 0.5 T SMF over seven days exerted the inhibitory effect on viability, proliferation, cytokine secretion, the expression of surface antigens and stem cell specific markers, and adipogenic and osteogenic differentiation but did not cause DNA damage in ASCs and _SPIO_ASCs.

## Figures and Tables

**Table 1:** Primers of specific markers for transdifferentiation, cytokines, and stem cell genetic markers.

Gene	Primer sequence	Fragment, bp	GenBank number
LPL			
Forward	5′CACCTACACACAAGCAAAGCCCCA3′	459	NM_012598
Reverse	5′GTGCTGTTCATCAGAGTGAGTTGGC3′		

PPAR-*γ*			
Forward	5′CTACACCATGCTGGCCTCCCTGATG3′	481	NM_013124
Reverse	5′TTGTCAGCGACTGGGACTTTTCTGC3′		

SPP1			
Forward	5′CGACAGTCAG GCGAGTTCCA AAGCC 3′	470	NM_012881
Reverse	5′GCTGTTCCTG TAAGTTTGCC TGCCTC 3′		

OSX			
Forward	5′AGTGGTGCAGGCAAACCTCCCCGGG 3′	405	BK00141
Reverse	5′GACCTGGCTCCCCGTGGGTGCGCTG 3′		

VEGF			
Forward	5′AGGTA CAGAG CAATG GGGCA GG 3′	689	NM_03836
Reverse	5′ACGGA ATATC TCGGA AAACT GCTC 3′		

IGF-1			
Forward	5′CATGT CGTCT TCACA TCTCT TCTAC 3′	299	NM_001082477
Reverse	5′CTTGT GTGTC GATAG GGGCT GGGAC 3′		

TGF-*β*1			
Forward	5′AAGGCTCGCCAGTCCCCCGA3′	400	NM_021578.2
Reverse	5′AGTGGGGGTCAGCAGCCGGT3′		

OCT4			
Forward	5′ ACCATCTGCCGCTTCGAGGC 3′	526	NM_001009178
Reverse	5′ TGGCTCACCTCATCCCCAGG 3′		

Sca1			
Forward	5′CCCGTGGACCCTGCCAGTGC 3′	761	X91619
Reverse	5′TGGCACCTGCGCACCCCTTC 3′		

ABCB1			
Forward	5′ GCCATGGCAGTGGGGCAGGT 3′	600	NM_133401
Reverse	5′ GATGCGCTGCTTCTGCCCGC 3′		

GAPDH			
Forward	5′ ATCTGACATGCCGCCTGGAGAAACC 3′	287	NM_017008
Reverse	5′ CAGGGTTTCTTACTCCTTGGAGGCC 3′		

LPL, Lipoprotein Lipase; PPAR-*γ*, Peroxisome Proliferator-Activated Receptor Gamma; SPP1, Secreted Phosphor-protein 1; OSX, Osterix; VEGF, Vascular Endothelial Growth Factor; IGF-1, Insulin-like Growth Factor-1; TGF-*β*1, Transforming Growth Factor Beta 1; Oct-4, Octamer-4; Sca1, Stem Cell Antigen-1; ABCB1, ATP-binding Cassette Subfamily B Member 1; GAPDH, glyceraldehyde-3-phosphate dehydrogenase.
